# Molecular imaging of atherosclerosis: spotlight on Raman spectroscopy and surface-enhanced Raman scattering

**DOI:** 10.1136/heartjnl-2017-311447

**Published:** 2017-10-23

**Authors:** Neil MacRitchie, Gianluca Grassia, Jonathan Noonan, Paul Garside, Duncan Graham, Pasquale Maffia

**Affiliations:** 1 Institute of Infection, Immunity and Inflammation, College of Medical, Veterinary and Life Sciences, University of Glasgow, Glasgow, UK; 2 British Society for Cardiovascular Research, UK; 3 Centre for Molecular Nanometrology, Department of Pure and Applied Chemistry, University of Strathclyde, Glasgow, UK; 4 Institute of Cardiovascular and Medical Sciences, College of Medical, Veterinary and Life Sciences, University of Glasgow, Glasgow, UK; 5 Department of Pharmacy, University of Naples Federico II, Naples, Italy

**Keywords:** cardiac imaging and diagnostics, aortic and arterial disease, carotid disease, peripheral vascular disease, inflammatory markers

## Abstract

To accurately predict atherosclerotic plaque progression, a detailed phenotype of the lesion at the molecular level is required. Here, we assess the respective merits and limitations of molecular imaging tools. Clinical imaging includes contrast-enhanced ultrasound, an inexpensive and non-toxic technique but with poor sensitivity. CT benefits from high spatial resolution but poor sensitivity coupled with an increasing radiation burden that limits multiplexing. Despite high sensitivity, positron emission tomography and single-photon emission tomography have disadvantages when applied to multiplex molecular imaging due to poor spatial resolution, signal cross talk and increasing radiation dose. In contrast, MRI is non-toxic, displays good spatial resolution but poor sensitivity. Preclinical techniques include near-infrared fluorescence (NIRF), which provides good spatial resolution and sensitivity; however, multiplexing with NIRF is limited, due to photobleaching and spectral overlap. Fourier transform infrared spectroscopy and Raman spectroscopy are label-free techniques that detect molecules based on the vibrations of chemical bonds. Both techniques offer fast acquisition times with Raman showing superior spatial resolution. Raman signals are inherently weak; however, leading to the development of surface-enhanced Raman spectroscopy (SERS) that offers greatly increased sensitivity due to using metallic nanoparticles that can be functionalised with biomolecules targeted against plaque ligands while offering high multiplexing potential. This asset combined with high spatial resolution makes SERS an exciting prospect as a diagnostic tool. The ongoing refinements of SERS technologies such as deep tissue imaging and portable systems making SERS a realistic prospect for translation to the clinic.

## Introduction

Atherosclerosis is a complex multifactorial pathology that progresses over many decades. Immune-inflammatory responses play key roles in all phases of the pathology and inflammation contributes to plaque rupture resulting in clinical manifestations such as myocardial infarction (MI) and stroke.^1^ The goal of contemporary researchers is to develop tools useful for the early diagnosis and accurate classification of those plaques which are stable and unlikely to be of immediate concern from those displaying a vulnerable phenotype and prone to rupture-thrombosis events.

Multiple imaging techniques are available for both invasive and non-invasive imaging of the vasculature. These include contrast-enhanced ultrasound (CEUS), CT, MRI, positron emission tomography (PET), single-photon emission tomography (SPECT), near-infrared fluorescence (NIRF) microscopy and vibrational spectroscopy. MRI, CT and PET/SPECT are already in clinical use for diagnosing and monitoring atherosclerosis and can provide structural information such as quantifying stenosis and plaque size/location. In some instances, invasive imaging using intra-arterial probes employing one of the above modalities can reveal basic morphological information on plaque composition, yet there remains an urgent unmet requirement for non-invasive imaging tools that can provide information beyond the anatomic and functional level that are able to interrogate the cellular and molecular pathways driving local inflammatory responses that promote plaque vulnerability.

Molecular imaging moves beyond anatomic and morphological detail to the level of cellular and molecular events. Activated endothelium offers a number of potential imaging targets, including adhesion molecules promoting leucocyte binding and transmigration into subendothelial locations. The complex inflammatory environment of a developing atherosclerotic plaque offers additional targets such as scavenger receptors present on macrophages/foam cells, and plaque destabilising proteases and peroxidases. True molecular imaging requires a targeting ligand (eg, antibodies, fragment antigen-binding (FAB), peptides, aptamers) to specific molecules/cells which are conjugated to a detection agent compatible with the imaging tool of choice, eg, microbubbles (CEUS), iodinated compounds (CT), radionucleotides (PET/SPECT), magnetic/superparamagnetic compounds (MRI) or fluorochromes (fluorescence imaging). Vibrational spectroscopy, such as Raman spectroscopy, offers the advantage of not requiring external imaging tracers owing to direct detection of endogenous biological molecules; however, Raman spectroscopy employing exogenous targeted nanoparticles (NPs) (surface-enhanced Raman spectroscopy (SERS)) results in an imaging capability with remarkably increased sensitivity and ability to simultaneously detect multiple analytes (multiplexing), which may improve diagnostic specificity. Advantages and limitations of the described techniques are summarised in table 1.

**Table 1:** Summary of non-invasive imaging modalities for use in atherosclerosis

Technique	Resolution	Scan time	Depth	Strengths	Limitations	Clinical use
CE-ultrasound	50 µM	Seconds–minutes	Several cms	Low cost, no radiation, high speed and sequential imaging, amenable to bedside testing	Poor sensitivity and signal-to-noise ratio make molecular imaging challenging, lack of vascular penetration confines information to endothelial surface	Plaque morphology, thrombus and ulceration detection, stenosis severity
CT	50 µM	Minutes	Body-wide	Relatively good spatial resolution	Low sensitivity, radiation exposure, signal cross talk between different tissue compartments	Plaque size, morphology, gross composition
PET/ SPECT	1–5 mm	Minutes	Body-wide	High sensitivity	Poor spatial resolution, radiation exposure, requires CT integration for anatomical analysis/quantification, limited multiplex potential due to signal cross talk and increasing radiation dose	Plaque inflammatory burden
MRI	10 µM–1 mm	Minutes–hours	Body-wide	Good spatial resolution, high soft tissue contrast, low toxicity imaging agents	Poor sensitivity, long imaging times often required, poor signal-to-noise ratio	Plaque inflammatory burden, morphology and stenosis severity
NIRF	1 µM–1 mm	Minutes	Several cms	Good spatial resolution and sensitivity, relatively low cost, no radiation, moderate multiplexing capability	Requires hybrid technologies for higher resolution imaging, photobleaching of fluorophores limits depth to superficial structures, relatively broad emission spectrum limits multiplexing, potential toxicity of imaging agents	None yet
FTIR	5–12 µM	Seconds–minutes	<1 cm	Label-free analysis, fast imaging times, broad classification of tissue morphology	Relatively low spatial resolution compared with Raman. Unable to discriminate closely related molecular structures	None yet
Raman	<1 µM	Seconds–minutes	Several mms	Label-free analysis, high spatial resolution, highly detailed classification of tissue morphology	Low sensitivity can prolong imaging times, poor signal-to-noise in some tissues, can require complex chemometric analysis to separate analytes	None yet
SERS	<100 nm	Seconds	Several cms	High sensitivity, low toxicity of gold nanoparticles, fast imaging times, no photobleaching, can use a broad spectrum of existing Raman dyes, deep tissue imaging possible with SESORS, strong multiplexing capacity, portable hand-held systems now available	Spectra are sensitive to changes in nanoparticle orientation and interference from adjacent structures, complex statistical algorithms will be required for separation and quantification of multiplexed spectra	None yet

In this review, we will highlight recent advances in molecular imaging and their application in identifying vascular inflammation, with a particular focus on the potentialities of Raman and SERS.

## Contrast-enhanced ultrasound

Ultrasound-based molecular imaging relies on CEUS, which employs the use of microbubbles as a vascular wall targeting agent. Signals are generated through vibration/oscillation or release of free gas when the microbubbles are exposed to sonic energy. Kaufmann *et al* 2 using microbubbles targeted to vascular cell adhesion molecule 1 (VCAM-1) and p-selectin demonstrated the feasibility of CEUS to detect endothelial vascular changes that preceded early lesion formation in mice, offering the possibility that CEUS could be in future used as an early screening tool for potential atherosclerosis development.

## Computed tomography

CT possesses relatively good temporal and spatial resolution which can further be improved on by multidetector spiral computed tomography and the concomitant use of iodinated contrast agents. This approach allowed the non-invasive identification of plaque location and size^3^ and the gross phenotypic classification of plaques as calcified, hypoechoic (soft) or hyperechoic (collagen-rich fibrous) with results correlating with those obtained by intracoronary ultrasound.^4^ Moreover, by employing multicolour (or spectral) CT, it is possible to distinguish between several composites including tissue calcium deposits and gold-labelled and iodine-labelled contrast agents allowing simultaneous imaging of multiple plaque components.^5^

As the current technology stands, clinical use of CT for cellular and molecular imaging of atherosclerosis may be restricted to the narrow use of identifying plaque mineral composition and cellular density. As with anatomic CT imaging, use in this area will be subject to careful review of the radioactive burden associated with CT X-ray exposure, especially in the context of repeated scans. To date, the low sensitivity of CT contrast agents combined with spatial resolution inadequate to detect vulnerable plaque components^6^ precludes its use for detailed molecular imaging of atherosclerotic plaques.

## Positron emission tomography/single-photon emission tomography

PET and SPECT are widespread imaging tools that rely on the administration of radionucleotides and subsequent detection of γ-rays to construct three-dimensional images of biological tissue forming the cornerstone of nuclear imaging. PET/SPECT are attractive options for molecular imaging owing to their non-invasive nature and high sensitivity. Furthermore, the high sensitivity of nuclear imaging is frequently combined with CT or MRI, tools with superior spatial resolution to form hybrid imaging systems such as PET/CT, SPECT/CT and PET/MRI.^7^

[18F]-Fluorodeoxyglucose (FDG) is a glucose analogue radiotracer commonly used in PET and uptake by tissues correlates with metabolic activity. Combined PET/CT imaging has shown that FDG accumulates more in symptomatic lesions than asymptomatic sites of stenosis, particularly in macrophage-rich regions.^8^ Since FDG is not specifically targeted to molecular events within the plaque, to try to improve the specificity of PET for vascular inflammation, new targeting ligands have been assessed in animal models. A VCAM-1 targeting peptide labelled with an 18-fluorine radiotracer was found to accumulate in the aortic root of atherosclerotic but not wild-type mice, being localised primarily to plaque endothelial cells.^9^ SPECT has also been used for in vivo imaging employing the common SPECT tracer technetium-99 bound to VCAM-1-targeted ligands.^10^ Dual-nucleotide micro-SPECT/CT imaging with a dual-head micro-SPECT scanner has been employed to assess the integration of distinct biological processes within the plaque, specifically matrix metalloproteinase activity and apoptosis^11^; however, issues of cross-talk between radionucleotide tracers are a complication in PET/SPECT multitarget imaging as is the enhanced toxicity risk of using agents producing multiple ionising radiation.

## Magnetic resonance imaging

MRI is perhaps the most studied tool to date for molecular imaging in atherosclerosis, owing to excellent spatial and temporal resolution without requiring the administration of ionising radioactive nucleotides.

To overcome the major weakness of MRI (poor sensitivity), the use of paramagnetic agents such as gadolinium (Gd) chelates and superparamagnetic (iron oxide microparticles and nanoparticles) contrast agents have been employed to non-invasively image arterial diseases. MRI molecular imaging of atherosclerosis primarily employs ultrasmall paramagnetic iron oxide particles (USPIOs; 10–50 nm in diameter). The properties of USPIOs allow extravasation into vascular tissues enabling potential analysis of atherosclerotic lesions. This approach has been used as a tool for measuring plaque macrophage burden through MRI signal loss following phagocytic uptake of USPIOs in rabbits^12^ and in humans where MRI signal intensity correlated with macrophage-rich vulnerable plaques.^13^ Technical issues associated with vessel wall imaging using USPIOs include low signal-to-noise ratio, poor specificity associated with the ability to clearly discriminate signal between anatomical locations, lengthy imaging times and the difficulty in standardising conditions between scans.^12^ Passive targeting of USPIOs shares the limitations of FDG used in PET in not being able to detect and define the molecular processes governing plaque progression. A more direct approach involved the use of peptides targeted against VCAM-1 conjugated to USPIOs which are taken up by inflamed endothelium in addition to macrophages and vascular smooth muscle cells, allowing the detection of fatty-streak lesions in juvenile hyperlipidemic apolipoprotein E (apoE)^-/-^ mice offering the potential for early intervention and monitoring.^14^

The use of microparticles of iron oxide (MPIO) offers several advantages over USPIOs. Their larger size (0.9–8 µm) confines their location to endothelial surfaces, preventing cellular uptake and potential consequences of cellular toxicity and loss of molecular specificity. Furthermore, the greater iron payload carried by MIOPs improves signal detection. These attributes coupled with rapid clearance from the circulation can allow early imaging of vascular inflammation with a high degree of specificity.^15^ One notable development is dual-antibody conjugated MPIOs targeted to p-selectin and VCAM-1, which display synergistically enhanced binding to plaque areas in the aortic root of apoE^-/-^ mice.^16^ Although the MPIOs employed in basic research studies precludes clinical use, the multitargeting of ligands will be a key feature in improving diagnostics for the accurate assessment of local inflammatory processes.

## Fluorescence imaging

Fluorescence imaging is an optical imaging technique that detects the emission spectra from fluorescent probes excited by a light source. As with other imaging modalities, probes are either passively or actively targeted to a biological molecule or tissue of interest or can act as ‘smart probes’ that are activated at the target site, for example, enzymatic conversion from a non-fluorescent substrate to a fluorescent product allowing functional read outs of local enzyme activity.^17^ Preclinical fluorescent imaging uses the near-infrared spectrum (wavelengths between 650 and 1000 nm) and is thus referred to as near-infrared fluorescence (NIRF) imaging. The advantages of imaging at these wavelengths is the lack of photonic absorbance from biological components allowing greater depth of imaging, and increased signal-to-noise ratio and hence specificity.^18^ Fluorophores are typically either proteins (eg, green fluorescent protein), small molecules or quantum dots (QDs). NIRF has been used to target markers of vulnerable plaques such as oxidised low-density lipoprotein-targeted fluorochrome-labelled antibodies^19^ and plaque destabilising proteases. Enzymatic targeting using enzyme substrates which fluoresce on proteolytic cleavage by a plaque containing protease have been employed ex vivo^20^ and in vivo in apoE^-/-^ mice.^21^ More recently, near-infrared autofluorescence has been proposed for the identification of intraplaque haemorrhage as a marker of high-risk atherosclerotic lesions in both mice and humans.^22^

The wide optical imaging window available for NIRF imaging allows the use of multiple probes to be assessed in a single system owing to fluorochromes emitting in distinct spectral regions; however, the difficulty in resolving such spectra often requires multiple excitation wavelengths for optimal signal discrimination.^23^ QDs are still constrained by relatively broad emission peaks making quantification of multiple low-level analytes problematic. Limiting factors restricting clinical use to date, include toxicity^24^ and loss of fluorescent signal.

## Vibrational spectroscopy

Vibrational spectroscopy mainly encompasses two techniques: infrared (IR) and Raman spectroscopies, which create molecularly specific spectra based on the vibrational energies of chemical bonds.

### Fourier transform infrared spectroscopy

FTIR, the most refined modern form of IR spectroscopy gains information from the vibration of molecular bonds caused by absorbance of light; requiring no external labelling method. The ability to detect chemically specific IR spectra over a wide wavelength window allows FTIR to detect multiple classes of biological molecules. FTIR is especially useful for differentiating lipids and proteins and these have been the main focus of FTIR research in cardiovascular studies to date.

FTIR has been employed to map the distribution of collagen I, collagen III and elastin in the fibrous cap region of human atheroma samples postmortem^25^ and the distribution of lipids, which can be used to map the location and morphology of plaques.^26^ Since the relative ratios of different lipid species within a plaque is also an indicator of the stage of the plaque, information discerned from FTIR could aid in determining the severity of a developing atheroma. At present, FTIR can discern compositional changes and aid in identifying unknown molecules in a tissue sample, although with relatively low spatial resolution in this regard. FTIR was found to be inferior to histological staining for quantifying lipid progression in advanced human atheroma^27^ and is outperformed by Raman spectroscopy in the detailed characterisation of plaque composition.^28^

## Raman spectroscopy

Like FTIR, Raman spectroscopy has been used to map the detailed composition of atherosclerotic plaques but improves on FTIR in terms of both spatial and spectral resolution.^29^ Importantly, stimulated Raman scattering under electronic preresonance conditions has recently been used to image up to 24 target molecules inside living cells, demonstrating the extensive multiplex capabilities of this technique.^30^ The potential of Raman spectroscopy to provide detailed information on plaque composition at micron and submicron resolutions has been examined in multiple studies summarised in table 2.^28 29 31–42^

**Table 2:** Research supporting the use of Raman spectroscopy as a molecular imaging tool in atherosclerosis

Tissue	Evidences	Species
Coronary artery	Gross plaque chemical composition to quantify cholesterol, cholesterol esters, triglycerides and phospholipids and calcium salts	Human; samples from 16 explanted recipient hearts; Raman analysis on sections^31^
	Detection of cholesterol	Human; intact artery samples^32^
	Quantification of relative proportions of multiple lipid classes including cholesterol, cholesterol esters, phospholipids and triglycerides	Human; arterial lysates^33^
	Detection and quantification of elastic laminae, collagen fibres, smooth muscle cells, adventitial adipocytes, foam cells, necrotic core, cholesterol crystals, β-carotene containing crystals and calcium mineralisation using an algorithm designed to interpret Raman data	Human; samples from 16 explanted recipient hearts; Raman analysis on 5μm thick sections^34^
	Relative quantification of cholesterol, collagen and adipocyte content	Human; fragments from 30 cadavers^35^
Brachiocephalic artery	Detailed Raman analysis of plaque content including lipids, remodelled media and fibrous cap with a spatial resolution down to 1 µM Observation of distinct protein signatures including haemoporphyrin and elastin	ApoE and LDLr DKO mice; transversal sections^28^
Aorta	Measurement of endothelial dysfunction	ApoE and LDLr DKO mice^36^
	Detailed Raman analysis of lesions associated with FTIR to characterise plaque contents including multiple lipid classes, endothelial cells, smooth muscle cells, extracellular matrix	Hypercholesteraemic rabbit; fragments^29^
	Quantitative mapping of chemical components	ApoE mice; fragments^37^
	Raman-probe spectroscopy to characterize the plaque composition of arterial walls	Rabbit; in vivo^38^
Carotid artery	Multimodal spectroscopic approach with the ability to detect vulnerable plaques with a sensitivity of 96%, specificity of 72% and a negative predictive value of 97%	Human; endarterectomy plus femoral bypass surgery samples (n=12)^39^
	Characterisation of peculiar spectral signatures related to biochemicals presented in lesion, eg, collagen and elastin, cholesterol and calcium hydroxyapatite	Human; fragments postmortem^40^
	Intravascular Raman spectroscopy, using miniaturised fibre-optic probes to collect Raman scattered light from vessel wall	Lambs and sheep; in vivo^41^
	Real-time in vivo collection of Raman spectra of atherosclerosis using a newly designed optical fibre Raman probe. Demonstration that Raman spectroscopy has capability to identify vulnerable plaque with 79% sensitivity and 85% specificity	Human^42^

Raman spectroscopy is a particularly attractive molecular imaging tool for interrogation of atherosclerotic plaques and potential diagnostic agent due to its ability to non-destructively acquire biochemical data on the processes that drive plaque development. Although the non-invasive nature of Raman is an important advantage of the technique, the main disadvantage pertains to its low sensitivity due to the weakness of the Raman scattering effect leading to long imaging times and an inability to discriminate low-level target molecules from background ‘noise’. Furthermore, many biological molecules are spectrally similar requiring complex chemometric analysis to separate signals of interest.

## Surface-enhanced Raman spectroscopy

The shortcomings associated with Raman spectroscopy have led to the development of SERS, a highly sensitive advancement of conventional Raman spectroscopy. The enhancement of Raman signal is achieved when a molecule is adsorbed onto or in close proximity to a metal surface and is dependent on the nature of metal surface, the excitation frequency used, the attachment of the molecule to the surface and also the nature of the molecule itself.^43^ Silver and gold provide excellent surface enhancement and can be used in a variety of physical formats including biocompatible NPs. Silver or gold NPs can be generated for use in vivo, functionalised with targeting molecules such as antibodies and tuned to work across a wide range of wavelengths from the visible to the near IR. This has given rise to a number of different applications of SERS in various scenarios including in vivo and in vitro diagnostic.^43^

**Figure 1:**
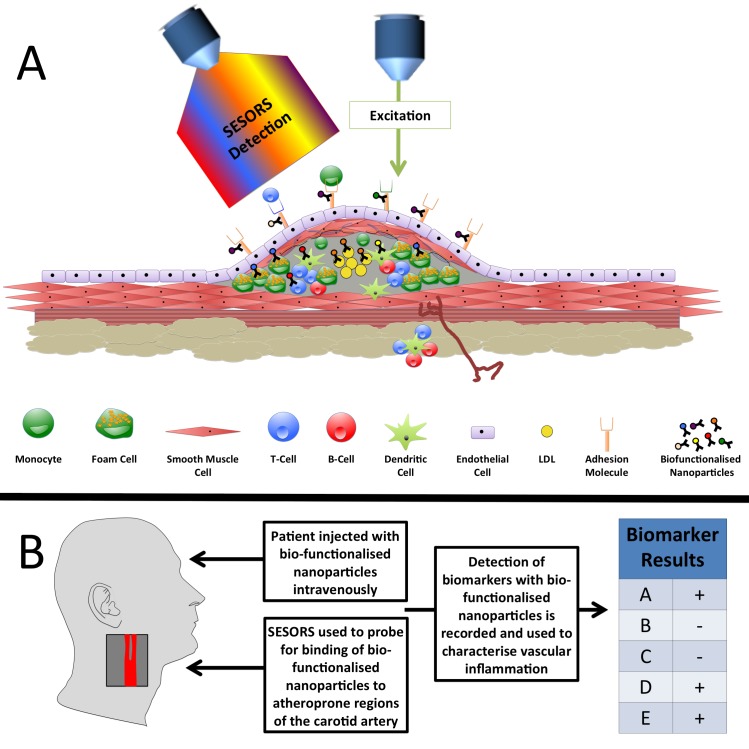
Prospective rational for surface-enhanced Raman spectroscopy (SERS) based-diagnostics for atherosclerotic inflammation. (A) The identification of inflammatory biomarkers in atherosclerotic plaques may be achieved via the intravenous injection of SERS-detectable biofunctionalised nanoparticles into a patient. Such nanoparticles would consist of a noble metal core for surface enhancement of Raman spectra; a Raman reporter to provide a predetermined Raman signature; a polymer coating (such as polyethylene glycol) to reduce nanoparticle interaction with the immune system and prolong blood circulation time; a targeting molecule such as an antibody or aptamer to confer molecular specificity. These nanoparticles would circulate in the vasculature, binding to inflammatory biomarkers such as adhesion molecules, macrophage, T-cell and B-cell activation markers and potentially atherosclerosis-associated biomarkers. Following binding these nanoparticles would then be detectable at significant tissue depths, eg, within the carotid artery, via surface-enhanced spatially offset Raman spectroscopy (SESORS). (B) Following the injection of biofunctionalised nanoparticles and the investigation of atheroprone regions of the vasculature with SESORS, detection of biomarker-specific biofunctionalised nanoparticles would be recorded. Consequently, this would provide clinicians with a technology capable of detecting a panel of biomarkers suitable for subsequent correlation with disease severity and implementation as a potential predictor of disease outcome. A hypothetical example is provided whereby the detection of biofunctionalised nanoparticles indicated the presence of biomarkers A, D and E, and the absence of biomarkers B and C following SESORS investigation of a carotid artery. LDL, low-density lipoprotein.

We have previously shown that intercellular adhesion molecule 1 (ICAM-1)-targeted NPs could detect ICAM-1 expression on aortic sinus tissue sections derived from apoE^-/-^ mice with a superior signal-to-noise ratio compared with immunofluorescence.^44^ Moreover, we supplemented this finding with in vivo data using an intradermal lipopolysaccharide challenge model in the ear pinna. Both fluorescence microscopy and SERS detected ICAM-1 expression in the ear vasculature but SERS showed superior sensitivity of detection.^44^ More recently, SERS gold nanorods were used for detection of adhesion molecules on inflamed macrophages.^45^ The ability to simultaneously detect multiple biomarkers (multiplexing) will be a key feature in early disease diagnosis. SERS multiplexing using five spectrally unique encoded NPs has been tested in vivo in mice following systemic administration^46^ with passive accumulation of non-targeted NPs observed in the liver at 24 hours postinjection. We are currently developing a SERS-based system for multiplexing detection of adhesion molecules ex vivo in human vascular samples and in vivo in mice in order to reveal early inflammatory changes in the vasculature (figure 1).^47^ Identifying the molecular signatures of atherosclerosis plaque in respect to multiple inflammatory markers could allow the interrogation of lesion staging and identify a vulnerable plaque phenotype.

### Therapeutic options coupled with SERS: photothermal apoptosis

The use of metallic NPs offers potential therapeutic applications to atherosclerosis, specifically through photothermal therapy in addition to acting as enhancers of Raman scattering. The photothermal effect arises when the plasmonic resonance wavelength of the NP matches the excitation wavelength of the laser and light is absorbed and converted to heat. If NPs are attached to or internalised by cells, the energy produced can be dissipated to the surrounding biological medium causing thermal destruction of the cell. Gold nanorods and single-walled carbon nanotubes are internalised by carotid artery resident macrophages in vivo following carotid ligation in mice and subsequent exposure of excised ligated vessels to NIR excitation-induced photothermal apoptosis of the macrophage population.^48^

The first human trial using plasmonic phototherapy, as a technique for regressing atherosclerosis, was the Plasmonic Nanophotothermal Therapy of Atherosclerosis (NANOM-FIM: https://clinicaltrials.gov/ct2/show/NCT01270139). This multicentre trial involved 180 patients aged 45–65 years diagnosed as having coronary artery disease with flow-limiting lesions. The two treatment groups received either a surgical implanted ‘on-artery’ patch capable of transferring silica-gold NPs to the plaque (Nano group) or an intracoronary infusion of silica-gold iron-bearing NPs with CD68 targeted microbubbles and stem cells via a magnetic navigation system (Ferro group). A control group received a stent implantation void of any NP. Following ‘detonation’ of the NPs via intravascular or transcutaneous NIR laser excitation, no adverse events related to the procedure such as site-related thrombosis were recorded throughout the 1-year follow-up. Both treatments were associated with a reduction in total atheroma volume and the nano group displayed increased survival relative to control during follow-up.^49^

Despite risks associated with induced apoptosis in the atheroma, this approach highlights the range of options coupled with SERS/NP usage. Gold NPs are also amenable as carriers for drug delivery to the vessel wall owing to their low toxicity, versatile surface chemistry conjugation and efficient uptake by vascular endothelial cells.^47^

## Conclusion and outlook

**Figure 2:**
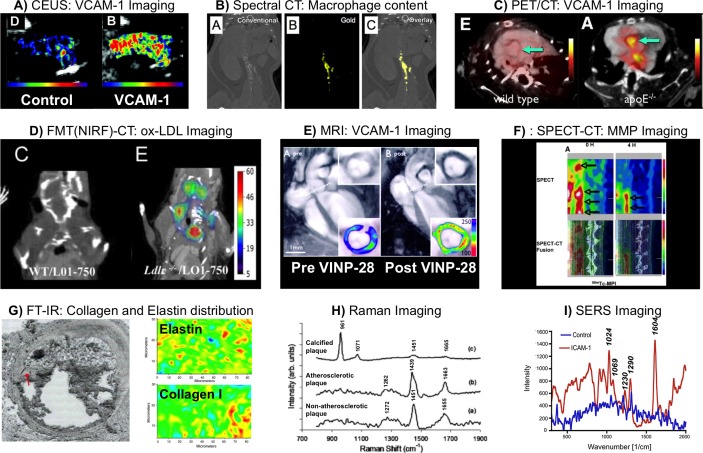
Examples of molecular imaging strategies for atherosclerotic disease. (A) Contrast-enhanced ultrasound (CEUS) molecular imaging using antibody functionalised biotinylated lipid shelled decafluorobutane microbubbles (MBs) in atherosclerotic mice. Comparison of isotype control (left panel) and antivascular cell adhesion molecule 1 (VCAM-1) (right panel) antibody functionalised MBs demonstrates high VCAM-1 expression in the aortic arch. Reprinted from Kaufmann *et al* 2, http://atvb.ahajournals.org/content/30/1/54.long (B) Spectral CT using high-density lipoproteins (HDL) labelled with gold has been used to image macrophage-rich plaques in apoE^-/-^ mice. Images displayed show conventional (left panel), spectral (middle panel) and overlay (right panel) CT, with enrichment for gold-HDL clear in the thoracic and abdominal aorta. Reprinted from Cormode *et al* 5, with permission from the Radiological Society of North America. (C) PET-CT imaging showing uptake of ^18^fluorine functionalised with a VCAM-1 targeting peptide (^18^F-4V) in the aortic root. Wild-type mice demonstrated low signal, while atherosclerotic apoE^-/-^ mice had significantly higher ^18^F-4V signal. Reprinted from Nahrendorf *et al* 9, with permission from Elsevier. (D) Using a fluorescently labelled anti-oxidised low-density lipoprotein (ox-LDL) antibody it was possible to image ox-LDL dense regions in atherosclerotic LDLr^-/-^ mice. No ox-LDL signal was detected from wild-type mice. Reprinted from Khamis *et al* 50, distributed under a Creative Commons CC-BY license. (E) Magnetic nanoparticles functionalised with a VCAM-1 targeting peptide (VINP-28) were used to image VCAM-1 using MRI. Atherosclerotic apoE^-/-^ mice were imaged pre-VINP-28 and post-VINP-28 injection. A marked signal drop was observed within the aortic root wall 48 hours postinjection. The contrast-to-noise ratio of the aortic wall was increased after the probe injection. Reprinted from Nahrendorf *et al* 14, http://circ.ahajournals.org/content/114/14/1504.long (F) Micro-single-photon emission tomography (SPECT) (top panels) and micro-SPECT-CT fusion images (bottom panels) for imaging a molecule binding to matrix metalloproteinases labelled with technetium-99m (Tc-MPI) in the rabbit atherosclerotic abdominal aorta, 0 and 4 hours after radiotracer administration. Reprinted from Haider *et al* 11, distributed under a Creative Commons Attribution Noncommercial License. (G) The fibrous cap of a plaque within a section of human coronary artery, identified by the red arrow (left panel), has been investigated using Fourier transform infrared spectroscopy (FTIR), demonstrating the ability to evaluate the distribution of elastin and collagen I (red/yellow=high; blue=low concentration). Reprinted from Wetzel *et al* 25, with permission from Elsevier. (H) Raman spectroscopy has been used to discriminate non-atherosclerotic tissue from atherosclerotic tissue, and also infer plaque phenotype, particularly with regard to calcified plaque. Shown here are Raman spectra of coronary arteries obtained ex vivo, highlighting the spectral differences between calcified and non-calcified atherosclerotic plaques in comparison with a non-atherosclerotic artery. Reprinted from Nogueira *et al* 40. (I) SERS spectroscopy of lipopolysaccharide-challenged ears show Raman peaks in mice with intravenous injections of anti-intercellular adhesion molecule 1 (ICAM-1)-conjugated nanotags (red) but no apparent spectra in mice with intravenous injections of control (IgG2b) nanotags (blue). Reprinted with permission from McQueenie *et al* 44. Copyright (2012) American Chemical Society.

Looking forward, this review has summarised activities in the common molecular imaging approaches in atherosclerosis, focusing on Raman and SERS. Images showing typical findings with each modality are shown in figure 2.^2 5 9 11 14 25 40 44 50^ The main area we see for advancement in the future is the development of highly specific particle probes for use in vivo with IR excitation and emission profiles to allow analysis at depth in tissue while retaining the molecular specificity of these techniques. Moreover, opportunities exist for the design of new NP composites, which will provide a therapeutic response including release of drugs. Many groups around the world appear to be starting to edge into this area and over the short term we expect there to be a large amount of visible activity and growth in this area coupled with some key lead examples which translate into clinical applications.
